# Mycobacterial antigen Ag85B restrains Hodgkin lymphoma tumor growth by inhibiting autophagy

**DOI:** 10.32604/or.2025.057842

**Published:** 2025-04-18

**Authors:** YONGFENG CHENG, YIPING SHEN, YUNFEI ZHANG, HAILIQIGULI NURIDING, XUEMEI WANG, CHUNYAN FAN, GULIBAHA MAIMAITI, YU LIU, YINGBIN YUE, DANLU LI, MEI YAN

**Affiliations:** 1 Department of Pediatrics, The First Affiliated Hospital of Xinjiang Medical University, Urumqi, 830054, China; 2 Division of Genetics and Genomics, Boston Children’s Hospital, Harvard Medical School, Boston, MA 02115, USA

**Keywords:** Hodgkin disease, Mycobacterial antigen, Autophagy, Protein kinase B, Mitogen-activated protein kinases

## Abstract

**Background:**

The growth of the B-cell lymphoma subtype, Hodgkin lymphoma (HL), is associated with increased autophagy. A mycobacterial antigen, Ag85, has been reported to inhibit cell autophagy under a variety of conditions. Whether Ag85 could inhibit autophagy in HL is unknown.

**Methods:**

Lymph node samples from patients with HL and healthy controls were collected to assess proliferation and autophagy. The human HL cell line, L-428, was cultured and subjected to Ag85B treatment. Autophagy in L-428 cells was evaluated through western blotting analysis, immunohistochemistry, and transmission electron microscopy. Apoptosis in these cells was measured using flow cytometry and western blotting. The associated signaling pathways were also analyzed utilizing western blotting. The *in vivo* impact of Ag85B was studied using BALB/c Nude mice xenografted with L-428 cells.

**Results:**

We observed increased proliferation and autophagy in primary lymphoma tissues of patients. Administration of Ag85B inhibited the proliferation and autophagy of HL cell lines. Moreover, Ag85B promoted apoptotic pathway activation *in vitro*, which might be associated with mitochondrial dysfunction. Mechanistically, Ag85B inhibits autophagy by activating the phosphatidylinositol-4,5-bisphosphate 3-kinase/protein kinase B/mechanistic target of rapamycin kinase (PI3K/AKT/mTOR) and mitogen-activated protein kinase (MAPK) pathways. Ag85B also inhibited lymphoma growth in mice xenografted with HL cell lines, but no potential toxicity was observed.

**Conclusion:**

Altogether, these results suggest that Ag85B inhibits HL growth via autophagy regulation. Current treatments for HL are associated with adverse events; therefore, Ag85B-mediated autophagy inhibition might be a promising strategy in to treat HL.

## Introduction

As a B-cell lymphoma subtype, Hodgkin lymphoma (HL) is categorized into nodular lymphocyte predominant HL (NLPHL) and classical HL (cHL) [1]. About 95% of HL is cHL, which features the presence of Hodgkin and Reed Sternberg (HRS) tumor cells [2]. The proportion of HRS tumor cells in the tumor mass is typically less than 1%, yet they exert a vital effect on HL pathogenesis [3]. Conventional treatment strategies for HL encompass hematopoietic stem cell transplantation, chemotherapy, and radiation therapy [4]. However, significant side effects are inevitable for these treatment strategies, which induce a high risk of toxicity, including secondary tumors, heart and lung toxicity, infertility, and fatigue. Given these challenges, there is a clinical imperative for treatments that offer high efficacy with reduced toxicity [5,6]. For this reason, and considering the multiple signaling pathways that are dysregulated in HL, targeting specific signaling pathways might be a novel option to improve HL treatment [3,7].

Basal autophagy, a critical cellular process that mediates the degradation and recycling of cellular components, has emerged as a complex player in tumorigenesis, with its role varying depending on the context. In the realm of solid tumors, basal autophagy is recognized for its dualistic nature, capable of promoting or suppressing tumor growth [8,9]. Moreover, reports suggest that dysregulated autophagy is a key factor in the pathogenesis of HL [10,11]. Studies have reported increased levels of autophagy-related proteins in HL, suggesting that heightened autophagy might be instrumental in sustaining the viability of malignant cells [12,13].

To date, some natural compounds have gained attention for cancer treatment, such as the inhibition of MCF7 breast cancer cells by nano-curcumin via suppression of cyclin D1 production, which affects breast cancer development and metastasis [14]. In addition, melatonin triggers autophagic cell death and inhibits the development of HL [15], highlighting the therapeutic potential of natural compounds in cancer treatment.

Mycobacterial antigens, such as ESAT-6, CFP10, and the Ag85 complex, are known to modulate immune responses, with the latter being highly conserved and existing as three distinct variants: Ag85A, B, and C [16,17]. In particular, Ag85B has been reported to inhibit autophagy in WI-38 cells within the context of tracheobronchial stenosis [18]. However, Ag85B’s function in the inhibition of autophagy of HL cells remains unexplored. Given the critical role of autophagy in tumor promotion and suppression, understanding the effects of Ag85B on HL could contribute to the development of novel therapeutic targets for HL.

Herein, we aimed to determine the impact of Ag85B on HL and to elucidate the underlying mechanisms, which might offer theoretical support to develop treatment strategies in HL. The investigation will focus on how Ag85B interacts with HL cells, potentially influencing autophagy-related proteins and signaling pathways that contribute to the pathogenesis of HL. By examining the effects of Ag85B on HL cells, we aim to increase our understanding of the complex interplay between mycobacterial antigens and cancer biology. Our findings might pave the way for new targeted therapies that leverage the modulation of autophagy as a strategy to combat HL, thereby improving patient outcomes and addressing the urgent need for more effective treatments.

## Methods

### Patient samples

Samples of tumor lymph nodes were collected from patients with cHL (n = 6). Control lymph nodes were obtained from normal lymph nodes from discarded tissues from patients (n = 6) who were determined to be free from all kinds of tumors. The research protocol was approved by the Institutional Review Board of The First Affiliated Hospital of Xinjiang Medical University (IRB, No., K202307-21). We performed these experiments based on ethical standards prescribed by the Helsinki Declaration. All patients provided informed consent.

### Mouse studies

A total of eight male and eight female BALB/c Nude mice (Cat. No., SM-014) (aged 6 to 8 weeks, body weight: 18–21 g) were obtained from Shanghai Model Organisms Center, Inc. (Shanghai, China), and housed in specific pathogen-free facilities at Xinjiang Medical University. The study was conducted with the approval (No. IACUC-JT-20240531-01) of the Animal Ethics Committee of Xinjiang Medical University. All mice were housed in barrier facilities with a 12 h light/12 h dark cycle, with *ad libitum* access to water and food. L-428 cells (3 × 10^7^ cells in 200 μL Roswell Park Memorial Institute (RPMI) 1640 medium without serum (Cat. No., C0893, Beyotime, Shanghai, China)) were inoculated subcutaneously into the nude mice’s flanks. Mice received Ag85B (40 mg/kg) via intraperitoneal injection and were monitored by measuring the tumor size weekly. The tumor diameter was detected and the tumor volume was calculated based on formula: 0.5 × length × width^2^. We monitored the mouse body weight weekly. The animals were anesthetized using carbon dioxide (CO_2_), and cervical dislocation was used to sacrifice mice when the tumor reached an appropriate volume. Tumor xenografts were then excised, weighted, stored, and fixed. To test the toxicity of Ag85 *in vivo*, mice in each group were randomly selected for dissection post-sacrifice, and the indicated visceral organs were stained with hematoxylin and eosin (H&E) to observe whether there were obvious abnormalities. Briefly, visceral organs samples were fixed in 4% paraformaldehyde, sectioned at 4 μm, stained using H&E (C0105S, Beyotime), and inspected using light microscopy (IX51 microscope; Olympus, Tokyo, Japan).

### Cell lines and treatments

We purchased the HL cell line L-428 from the German Collection of Microorganisms and Cell Cultures (DSMZ, Braunschweig, Germany). We cultured the cells in RPMI-1640 medium containing 10% fetal bovine serum (Cat. No., C0226, Beyotime) and 100 U/mL penicillin/streptomycin (Cat. No., C0222, Beyotime) in an incubator under standard conditions. All cells were free of mycoplasma contamination. The cells were treated with 0, 1, 2, 4, or 8 μg/mL of tuberculosis (TB) major secretory protein antigen 85B (Ag85B, bs-41043P, Bioss, Beijing, China). Lysosomal inhibitors E64d (Cat. No., E8640, Sigma, St. Louis, MO, USA) and pepstatin A (PepA, Cat. No., 516483, Sigma) (both at 10 μg/mL) were used to treat the indicated cells.

### Morphological analysis and immunohistochemistry

The tumor samples were cut into 4 µm slices and stained with H&E or the indicated antibodies. For H&E staining, tissues were fixed using 4% paraformaldehyde for 24 h, followed by paraffin embedding and sectioning at 4 μm thickness. Subsequently, the sections were dehydrated using a gradient alcohol series, cleared, and mounted. The sections were then subjected to H&E staining. Ultimately, tissue injury was assessed under a light microscope (IX51; Olympus). For immunohistochemistry, tissues embedded in paraffin were sectioned into 4-micron-thick slices. The tissue sections underwent a series of washing steps: they were first deparaffinized three times in xylene to remove paraffin, followed by two washes with anhydrous ethanol, two washes with 90% ethanol, one wash with 70% ethanol, and finally two washes with double distilled water to complete the preparation process. Following rehydration, the sections were subjected to antigen retrieval via heating in a microwave, and then rinsed with phosphate-buffered saline (PBS) for 5 min. Subsequently, the sections were incubated with 3% hydrogen peroxide (H_2_O_2_) for 25 min. After another round of washing with PBS, the sections were incubated with goat serum for 30 min to block non-specific binding. Primary antibodies were applied to stain the samples overnight at 4°C. Next day, the sections were incubated with a horseradish peroxidase (HRP)-labeled secondary antibody for 30 min. 3,3′-diaminobenzidine (DAB, P0202, Beyotime) was applied to develop the color. Positive cellular staining was visualized under a microscope (IX51; Olympus). Terminal deoxynucleotidyl transferase nick-end-labeling (TUNEL) staining was applied to detect tumor apoptosis and necrosis using a TUNEL assay kit (Cat. No., C1091, Beyotime) following the supplier’s guidelines. Briefly, the samples underwent fixation and permeabilization, followed by inactivation of endogenous peroxidases using 0.3% hydrogen peroxide (H_2_O_2_) for 20 min. Thereafter, the samples were incubated with the TUNEL reagent at 37°C for 60 min. Nuclear staining was achieved using DAB for 10 min, followed by hematoxylin counterstaining. Ultimately, light microscopy (IX51; Olympus) was used to observe and identify apoptotic cells. ImageJ (version, 1.51j, NIH, Bethesda, MD, USA) was then employed to analyze the images. The primary antibodies used are listed in Table 1.

**Table 1:** Source and identifier of antibodies used in this study

Antibodies	Source	Identifier	Dilution ratio
LC3B	Affinity, OH, USA	AF4650	WB 1:1000; IHC 1:100
Beclin-1	Affinity, OH, USA	AF5128	WB 1:1000; IHC 1:50
p62	Affinity, OH, USA	AF5384	WB 1:1000; IHC 1:100
Lamp1/2	Affinity, OH, USA	DF4806	WB 1:500; IHC 1:100
PINK-1	Affinity, OH, USA	DF7742	WB 1:1000; IHC 1:100
Park2	Affinity, OH, USA	AF0235	WB 1:1000; IHC 1:100
ULK1/2	Affinity, OH, USA	DF7588	WB 1:1000; IHC 1:100
Cleaved caspase-3	Affinity, OH, USA	AF7022	WB 1:1000; IHC 1:100
Goat Anti-Rabbit	Abcam, Cambridge, UK	ab205718	IHC 1:10000
Goat Anti-Mouse	Abcam, Cambridge, UK	ab205719	IHC 1:10000
Atg14	Affinity, OH, USA	AF7912	WB 1:1000
β-actin	Proteintech, Chicago, USA	20536-1-AP	WB 1:5000
Cyclin D1	Abcam, Cambridge, UK	ab134175	WB 1:10000
CDK1	Affinity, OH, USA	DF6024	WB 1:1000
CDK2	Affinity, OH, USA	AF6237	WB 1:1000
Cyclin E1	Affinity, OH, USA	AF0144	WB 1:1000
Cyclin A2	Abcam, Cambridge, UK	ab32386	WB 1:10000
Cyclin B2	Abcam, Cambridge, UK	ab185622	WB 1:1000
c-MYC	Affinity, OH, USA	BF8036	WB 1:1000
p53	Affinity, OH, USA	BF8013	WB 1:1000
p21	Affinity, OH, USA	AF6290	WB 1:1000
Cleaved caspase-8	Affinity, OH, USA	AF5267	WB 1:1000
Caspase-7	Affinity, OH, USA	DF6441	WB 1:1000
Caspase-3	Affinity, OH, USA	AF6311	WB 1:1000
DR5	Affinity, OH, USA	DF6368	WB 1:1000
PARP	Abcam, Cambridge, UK	Ab139417	WB 1:500
Cleaved caspase-9	Affinity, OH, USA	AF5244	WB 1:1000
Cytochrome C	Zenbio, Chengdu, China	r24044	WB 1:500
Bax	Affinity, OH, USA	AF0120	WB 1:1000
PUMA	Abcam, Cambridge, UK	ab9645	WB 1:10000
Bcl-xL	Zenbio, Chengdu, China	380188	WB 1:500
Cleaved-PARP	Affinity, OH, USA	AF7023	WB 1:1000
PI3K	Zenbio, Chengdu, China	380849	WB 1:500
p-PI3K	Zenbio, Chengdu, China	341468	WB 1:500
Akt	Zenbio, Chengdu, China	342529	WB 1:500
p-Akt	Zenbio, Chengdu, China	310021	WB 1:500
mTOR	Zenbio, Chengdu, China	380411	WB 1:500
p-mTOR	Zenbio, Chengdu, China	381548	WB 1:500
ERK1/2	Zenbio, Chengdu, China	343830	WB 1:500
p-ERK1/2	Zenbio, Chengdu, China	310065	WB 1:500
p38	Zenbio, Chengdu, China	200782	WB 1:500
p-p38	Zenbio, Chengdu, China	310068	WB 1:500
GSK3β	Zenbio, Chengdu, China	221162	WB 1:500
p-GSK3β	Zenbio, Chengdu, China	310010	WB 1:500

### Proliferation

Cell proliferation was assessed employing Cell counting kit-8 (CCK-8; Cat. No., C0037, Beyotime) assays following the supplier’s guidelines. For cell viability assessment, cells in 200 μL of medium were added into the wells of a 96-well plate at 0.5 × 10^4^ cells per well. Subsequently, varying concentrations of Ag85B were introduced to the wells. At 12, 24, and 48 h post-treatment, each well received 10 μL of CCK-8 solution and incubated in the dark for 120 min. A microplate reader (Tecan Infinite F50, Männedorf, Switzerland) was then employed to measure the absorbance of each well at 450 nm. The following formula was utilized to calculate the cell survival rate: Cell Viability Rate (%) = (A_EX_ − A_OE_)/(A_NC_ − A_OE_) × 100%. Where A_EX_ is the experimental group absorbance, A_OE_ is the optical empty (blank) absorbance, and A_NC_ is the negative control absorbance.

### Colony formation assay

Methyl cellulose colony assay solution was mixed in an equal amount with HL cells and inoculated into a 12-well plate at 1 × 10^4^ cells per well, and cultured for 10 days. Colonies with a diameter greater than 0.1 mm were counted.

### Detection of Reactive oxygen species (ROS)

Cellular ROS were detected employing a dichloro-dihydro-fluorescein diacetate (DCFDA) cellular ROS detection assay kit (ab113851, Abcam, Cambridge, MA, USA) based on the supplier’s instructions. DCFDA (20 μM in 1× buffer) was incubated with the collected cells for 30 min at 37°C to stain for ROS. Directly after staining, without interposing a washing step, the cells were analyzed using flow cytometry (fluorescence activated cell sorting (FACS)) on a FACS LSRFortessa instrument (BD Biosciences, San Jose, CA, USA; excitation wavelength = 488 nm, emission wavelength = 535 nm). Each measurement was determined according to the average fluorescence intensity of 10,000 cells.

### Autophagic flux analysis (mRFP-GFP-LC3 assay)

Autophagosomes, autolysosomes, and autophagic flux were detected using mRFP-GFP-LC3B adenovirus (mRFP, monomeric red fluorescent protein; GFP, green fluorescent protein; LC3B, microtubule associated protein 1 light chain 3 beta; HANBIO, Shanghai, China) transduction based on the manufacturer’s instructions. At 24 h after transduction, the cells were cultured in a suitable environment for another 24 h. Thereafter, fluorescence microscopy (LSM880, Zeiss, Baden-Württemberg, Germany) was used to detect the fluorescence. In the detection results, the yellow (overlap of mRFP and GFP) and red (mRFP only) fluorescent dots represented early autophagosomes and autolysosomes, respectively.

### Mitochondrial mass detection

Mitochondrial mass was detected employing MitoTracker Green FM (Cat. No., M46750, Thermo Fisher Scientific, Waltham, MA, USA) according to the supplier’s guidelines. Cells were resuspended in PBS and subjected to 40 nM MitoTracker green (MTG) staining for 15 min at 37°C. The stained cells were then analyzed using flow cytometry (BD Biosciences).

### Transmission electron microscopy

Ag85B was used to treat L-428 cells for 24 h and then the cells were processed for transmission electron microscopy (TEM; JEM-1200 transmission electron microscope (JEOL, Tokyo, Japan)) as described previously [19].

### ATP assay

Based on the supplier’s instructions, we applied an ATP assay kit (Cat. No., 11699695001, Roche, Basel, Switzerland) to measure intracellular ATP levels. Collected cells were heated in a test tube at 100°C for 7 min. After centrifugation at 12,000× *g* for 5 min, the supernatant was transferred to the wells of a 96-well plate and luciferase solution was added to each well in the dark. Luminescence was then detected.

### Cell cycle and apoptosis analysis

A Cell Cycle assay kit (Cat. No., CA1510, Solarbio, Beijing, China) was used for cell cycle analysis. An Annexin V-Phycoerythrin (PE) Apoptosis Detection Kit (Cat. No., C1065M, Beyotime) was applied for apoptosis analysis. Both kits were used according to their manufacturers’ instructions.

### Analysis of mitochondrial membrane potentials in HL cells

The mitochondrial membrane potential (MMP) was detected using a JC-1 Staining Dye Assay Kit (Cat. No., M8650, Solarbio). Fluorescence microscopy and flow cytometry were used to analyze the cells.

### Western blotting

Radioimmunoprecipitation assay (RIPA) Lysis Buffer (P0013C, Beyotime) was used for homogenization of samples from each group at 4°C. Following homogenization, the samples were centrifuged at 1000× *g* for 10 min. The protein concentrations in the retained supernatant were assessed employing a Bicinchoninic Acid (BCA) Protein Assay Kit (Cat. No., P0010, Beyotime). Subsequently, the protein samples were electrophoresed through a 10% SDS-PAGE gel and then transferred electrophoretically onto polyvinylidene difluoride (PVDF, HVLP04700, Millipore, Billerica, MA, USA) membranes. After blocking with 5% bovine serum albumin (BSA, ST023, Beyotime), primary antibodies were added and incubated with the membranes at 4°C overnight. Then, following incubation with HRP-conjugated secondary antibodies, ImageJ software was used to visualize and quantify the immunoreactive protein bands. The primary antibodies are listed in Table 1.

### Immunofluorescence

Cells were harvested and fixed with in 4% paraformaldehyde for 30 min. After fixation, the cells were rinsed twice with PBS and then permeabilized and blocked with 5% bovine serum albumin (BSA) for 30 min. Subsequently, the cells were incubated with an anti-Ki-67 antibody (Cat. No., 27309-1-AP, Proteintech, Wuhan, China) at dilution of 1:10000 overnight at 4°C in the dark. Subsequently, secondary antibodies (Cat. No., SA00007-2, Proteintech, dilution ratio, 1:100) were added to the cells and incubated in the dark for 60 min at room temperature. Finally, the samples were mounted with 4′,6-diamidino-2-phenylindole (DAPI) (Cat. No., C1005, Beyotime) and examined using an inverted confocal microscope (LSM880, Zeiss). The relative fluorescence intensity was quantified using ImageJ software.

### Statistical analysis

The results are presented as the mean ± SEM, assuming a normal distribution of the data. Data normality was verified employing the Kolmogorov–Smirnov test. Homogeneity of variance was assessed utilizing Levene’s test. Differences between groups were determined using Student’s *t*-test and analysis of variance (ANOVA) followed by Tukey’s multiple comparison test. All data were processed using GraphPad Prism 9 software (GraphPad Inc., La Jolla, CA, USA). Statistical significance was accepted at the level of 0.05.

## Results

### Increased proliferation and autophagy in HL

**Figure 1:**
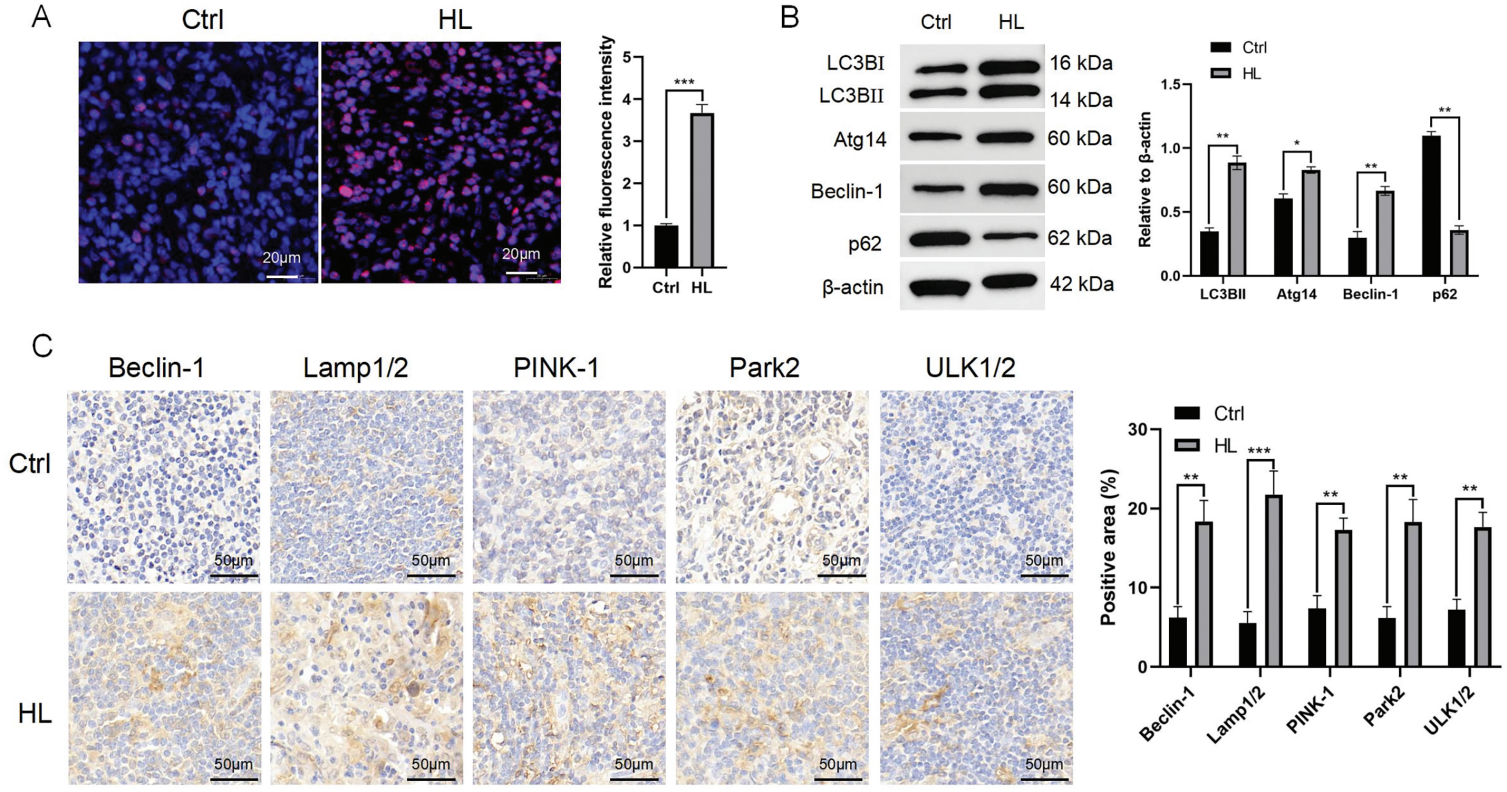
Proliferation and autophagy are increased in HL. A. Ki-67 staining of lymphoma primary tissue (patients with HL) compared with normal lymphoid tissue (non-malignant tumor patients). Scale bars = 20 μm. B. The levels of LC3B, Atg14, Beclin-1, and p62 in lymphoma primary tissue compared with those in normal lymphoid tissue were assessed employing western blotting. Right panel: densitometry analysis. C. Immunohistochemical staining of ULK1/2, Park2, PINK-1, Lamp1/2, and Beclin-1 in lymphoma primary tissue and normal lymphoid tissue. Scale bar = 50 μm. Data are expressed as the mean ± SEM from two independent experiments (n = 6). *p < 0.05, **p < 0.01, ***p < 0.001. Ctrl, control; HL, Hodgkin lymphoma; Ki-67, marker of proliferation Ki-67; LC3B, microtubule associated protein 1 light chain 3 beta; Atg14, autophagy related 14; p62, sequestosome 1; lamp, lysosomal associated membrane protein; PINK-1, PTEN induced kinase 1; parkin RBR E3 ubiquitin protein ligase, Park2; ULK, Unc-51 like autophagy activating kinase.

Excessive of proliferation is the basic property of tumors. A marked increase in the proportion of Ki-67-positive cells in lymphoma primary tissue was observed compared with that in control tissue (Fig. 1A).

In cancerous tissue, there is often a significant increase in levels of autophagy-related proteins. Thus, we detected key autophagy proteins using western blotting in lymphoma primary tissue and control tissue. Compared with those in control tissue, lymphoma showed significant increases of in LC3B (LC3BI represents early autophagy, while LC3BII represents autophagic flux), autophagy related 14 (Atg14) and Beclin-1 levels, and a decrease in p62 (sequestosome 1) levels (Fig. 1B).

We additionally conducted immunohistochemical staining in lymphoma cells. HL cells showed more extensive positivity for Beclin-1, Unc-51 like autophagy activating kinase (ULK)1/2, parkin RBR E3 ubiquitin protein ligase (PRKN or Park2), PTEN induced kinase 1 (PINK-1), and lysosomal associated membrane protein (Lamp)1/2 than did the controls (Fig. 1C).

### Ag85B inhibits autophagy in human HL cells

**Figure 2:**
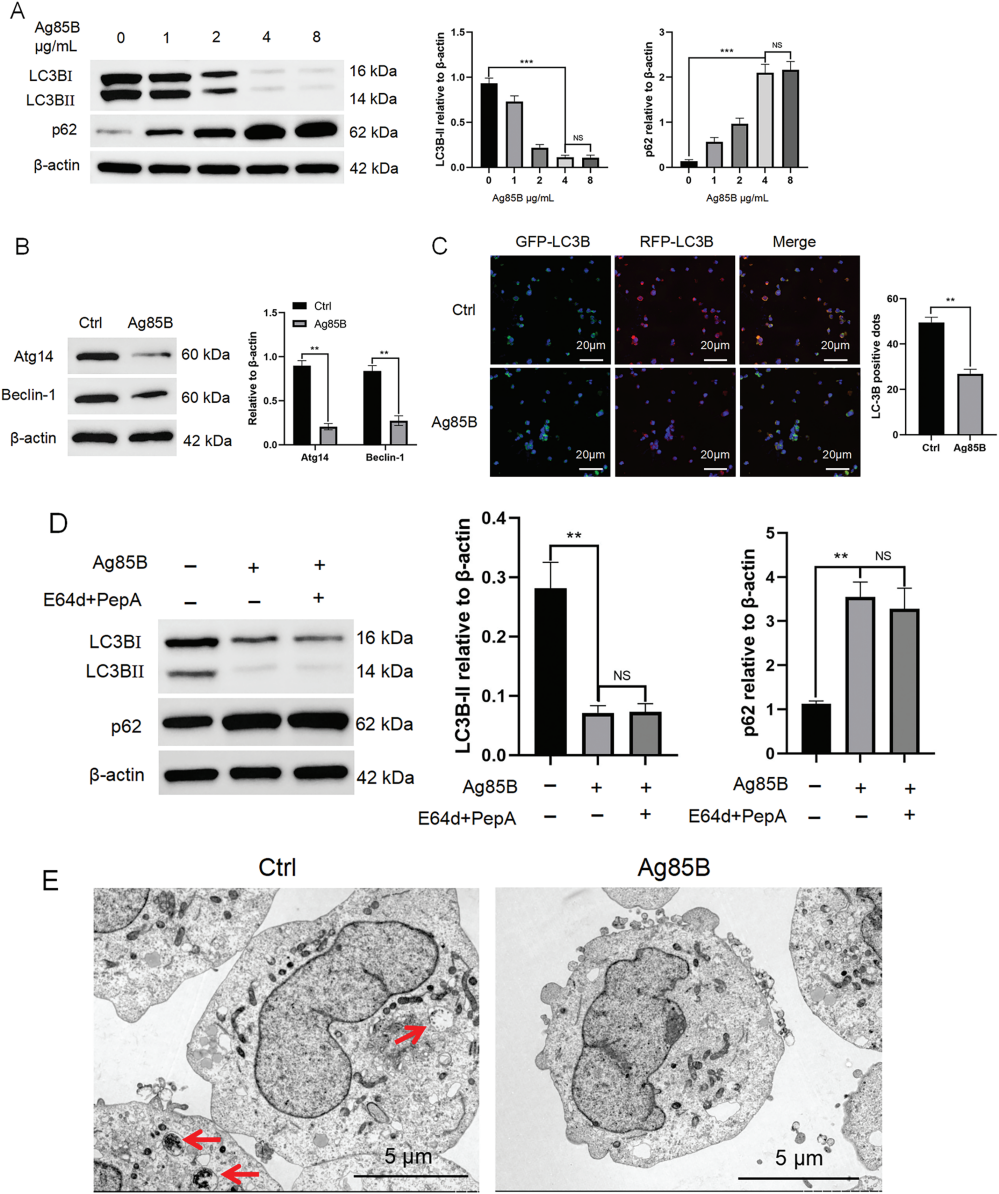
Ag85B inhibits autophagy in L-428 cells. A. Levels of LC3B and p62 proteins measured using western blotting in L-428 cells treated with Ag85B or vehicle. B. Western blotting for the levels of Beclin-1 and Atg14 in L-428 cells administered with Ag85B or vehicle. C. Fluorescence microscopy was utilized to detect the level of mRFP+/GFP+(yellow) and mRFP+/GFP-(red) LC3 puncta in HL cells treated/not treated with Ag85B (4 μg/mL) for 24 h. Typical images and fluorescent LC3 puncta quantitative analysis are shown. Scale bars = 20 μm. D. Immunoblotting assessment of the levels of p62 and LC3 in lysates from L-428 cells treated/not treated withAg85B for 24 h, with or without E64d and PepA (both at 10 μg/mL; sigma). E. TEM observation of autophagosomes in L-428 cells treated/not treated with Ag85B. The autophagosome is shown by an arrow. Scale bars = 5 μm. Data are shown as the mean ± SEM from 2–3 independent tests (n = 6). **p < 0.01, ***p < 0.001, NS, no significance. Ctrl, control; HL, Hodgkin lymphoma; Atg14, autophagy related 14; p62, sequestosome 1; RFP, red fluorescent protein; LC3, microtubule associated protein 1 light chain 3; E64d, cysteine protease inhibitor; PepA, pepstatin A; TEM, transmission electron microscopy.

Autophagy, a critical cellular process implicated in the etiology and progression of neoplastic diseases, has garnered significant attention in oncological research. Our investigation builds upon a seminal study that reported the inhibitory effect of Ag85B (2 μg/mL) on autophagy in tracheobronchial stenosis [19]. With the aim of elucidating the regulatory role of Ag85B in autophagy of HL L-428 cells, a well-characterized lymphoma cell line, we conducted a systematic analysis of autophagy markers, specifically LC3B and p62. In our experimental paradigm, cells were treated with a gradient of Ag85B concentrations ranging from 1 to 4 μg/mL, and the autophagy markers were assessed using immunoblotting. The results revealed a significant decrease in LC3B levels and a concomitant increase in p62 levels in an Ag85B dose-dependent manner, suggesting that Ag85B modulates autophagy in a concentration-dependent fashion. Notably, the autophagy markers did not exhibit further alterations upon exposure to Ag85B concentrations exceeding 4 μg/mL, indicating a potential threshold effect (Fig. 2A). Thus, subsequent experiments used Ag85B at 4 μg/mL. Western blotting verified that Ag85B decreased the levels of Atg14 and Beclin-1 (Fig. 2B). To investigate whether Ag85B could reduce HL cell autophagic flux, HL cells were transduced with mRFP-GFP-LC3B adenovirus and autophagy was assessed. In this assay, yellow dots represent autophagosomes and red dots represent autolysosomes. On this basis, Ag85B was added and incubated for 24 h, and fluorescence microscopy was used to acquire images. The numbers of mRFP+GFP− (red) autolysosomes were significantly decreased in HL cells treated with Ag85B (Fig. 2C), suggesting that Ag85B inhibited autophagy.

To fully explore this mechanism, we also used the lysosomal inhibitors E64d and PepA. Essentially, LC3B levels can be reduced because of enhanced downstream autophagosome lysosomal fusion or reduced upstream autophagosome formation. To clarify the specific reasons, we detected the Ag85B-induced LC3B accumulation with and without autophagy inhibitors. After treatment with E64d and PepA, Ag85B-induced autophagy markers did not change significantly (Fig. 2D), being a consequence of decreased upstream autophagosome formation.

To further investigate the issue of autophagy induction, we used TEM to study the ultrastructure of L-428 cells in detail. The presence of cell debris, partially degraded endoplasmic reticulum, and autophagic vacuoles was observed in control cells, but not in Ag85B-treated L-428 cells (Fig. 2E). Together, these results indicated that Ag85B inhibits autophagy in HL.

### Ag85B inhibits HL cell proliferation and cell cycle progression

**Figure 3:**
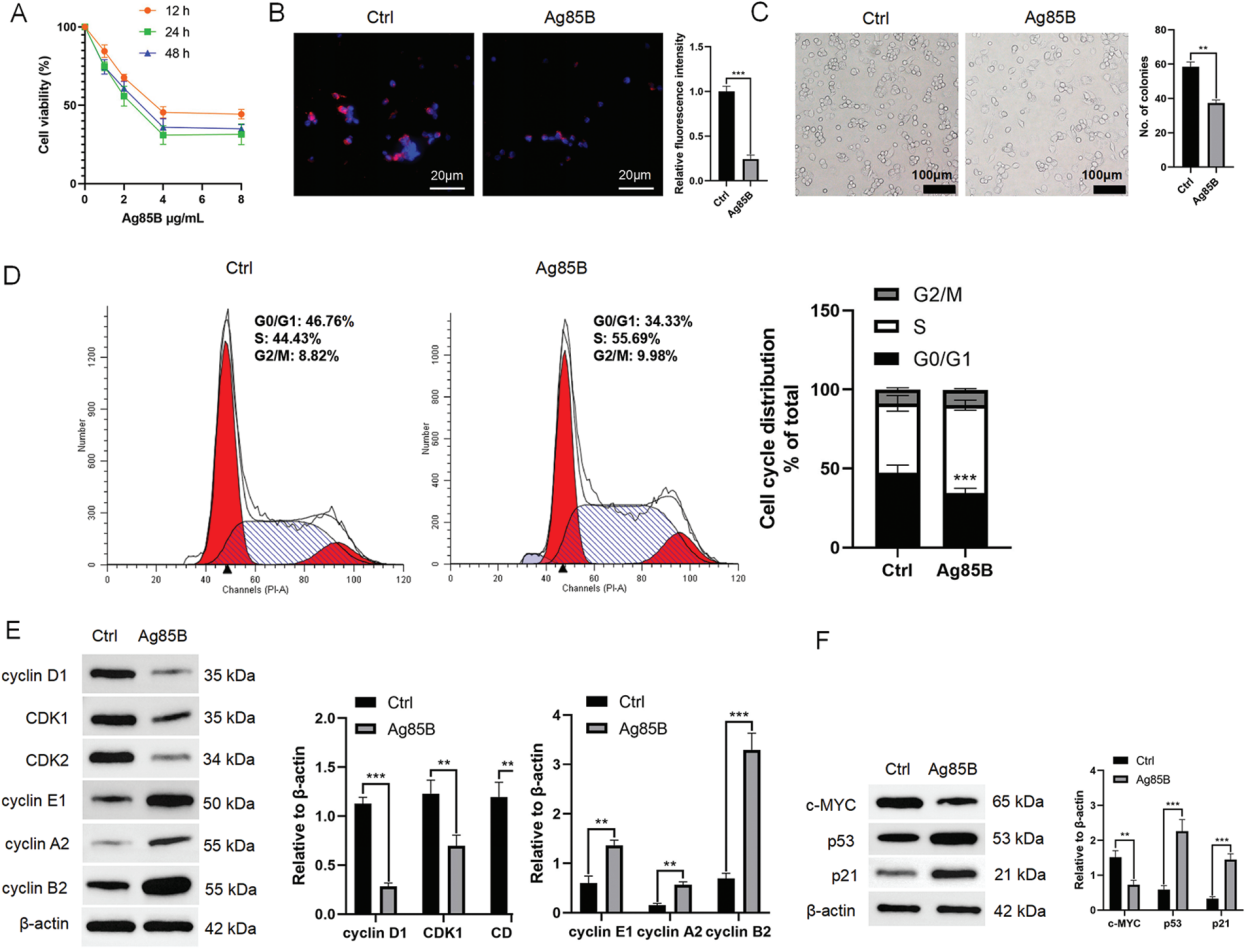
Ag85B inhibits HL cell proliferation by preventing the cell cycle. A. Viability of L-428 cells treated with different doses of Ag85B for 24 h, and detected via a CCK-8 assay. B. Ki-67 staining in L-428 cells. Scale bars = 20 μm. C. A colony formation assay was employed to assess cell proliferation. Scale bars = 100 μm. D. Flow cytometry detection of the progression of the cell cycle in L-428 cells. E, F. Immunoblotting detection of p21, c-MYC, CDK, and cyclin levels in L-428 cells. Data represent the mean ± SEM from 2–3 independent experiments (n = 6). **p < 0.01, ***p < 0.001. Ctrl, control; HL, Hodgkin lymphoma; CCK-8, cell counting kit 8; Ki-67, marker of proliferation Ki-67; CDK, cyclin dependent kinase; c-MYC, MYC proto-oncogene, BHLH transcription factor; p21, cyclin dependent kinase inhibitor 1A; p53, tumor protein p53.

We next explored whether Ag85B treatment affects cell proliferation in human HL cell lines. We examined relative cell viability in cells treated with different concentrations of Ag85B. Ag85B concentrations at 1 to 4 μg/mL decreased cell viability in a dose-dependent manner; however, cell viability was not affected by further increases the concentration of Ag85B (Fig. 3A). After treatment with Ag85B, the proportion of Ki-67 positive HL cells decreased significantly (Fig. 3B). We also detected whether Ag85B affected the long-term survival of L-428 cells. There was a significant reduction in L-428 colony numbers after treatment with Ag85B (Fig. 3C).

Ag85B administration also significantly reduced the G0/G1 phase cell population and significantly increased the number of S phase cells. There was no significant alteration in the number of G2 phase cells (Fig. 3D). The regulatory mechanism of the cell cycle is very complex and is the result of the combined action of many proteins, such as cyclins and cyclins-dependent kinases (CDKs) [20]. After 24 h of treatment with Ag85B, the levels of cyclin D1, CDK1, and CDK2 were downregulated in HL cells, while cyclin E1, cyclin A2, and cyclin B2 levels were increased (Fig. 3E). These results suggested that Ag85B induces S phase arrest and regulates the expression of G1/S phase-related proteins. MYC proto-oncogene, BHLH transcription factor (c-MYC) plays a very important role in the cell cycle, and its mechanism of action is mainly achieved by regulating the expression of cyclin dependent kinase inhibitor 1A (CDKN1A or p21) and tumor protein p53 (p53) [21]. Ag85B also decreased the level of c-MYC and raised p53 and p21 levels (Fig. 3F). These results indicated that Ag85B strongly inhibited cell proliferation.

### Ag85B induces HL cells apoptosis

**Figure 4:**
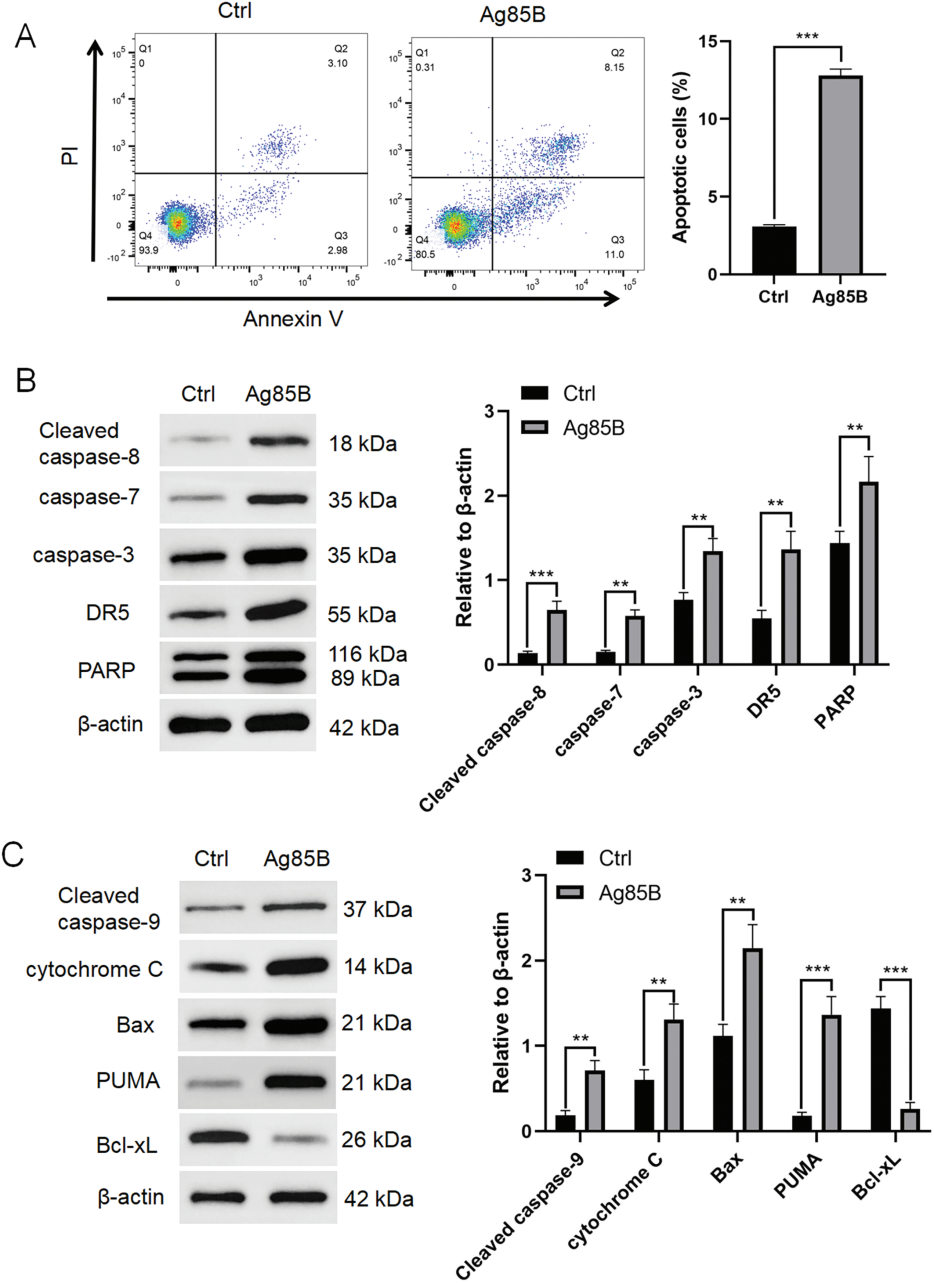
Ag85B induces cells apoptosis. A. Apoptosis in L-428 cells as assessed using flow cytometry. The percentage of Annexin+/PI- cells represents apoptotic cells; Annexin+/PI+ represents late apoptotic or necrotic cells. B, C. Levels of apoptosis-associated proteins were measured via immunoblotting in L-428 cells. Data represent the mean ± SEM from 2–3 independent experiments (n = 6). **p < 0.01, ***p < 0.001. Ctrl, control; PI, propidium iodide; PARP, poly(ADP-ribose) polymerase; DR5, death receptor 5; Bax, Bcl2 associated X; PUMA, p53 up-regulated modulator of apoptosis; Bcl-xl, Bcl-2-like protein 1.

We also explored other mechanisms through which Ag85B inhibits HL cell growth. Among HL cells, early and late apoptosis or necrotic cells increased significantly after Ag85B treatment (Fig. 4A). Apoptosis can be initiated by the extrinsic or intrinsic pathway. The induction of cell apoptosis triggers the activation of caspase-8 (extrinsic pathway) or caspase-9 (intrinsic pathway) [22]. Ag85B induced the cleavage of caspase-8 and its downstream targets, caspase-7 and caspase-3, death receptor 5 (DR5), and poly (ADP-ribose) polymerase (PARP) (Fig. 4B). Furthermore, Ag85B regulated B-cell CLL/lymphoma 2 (Bcl-2) expression and the levels of mitochondrial damage-related proteins. The levels of cleaved caspase-9, cytochrome C, Bcl2 associated X (Bax), and p53 upregulated modulator of apoptosis (PUMA) were upregulated, while the level of antiapoptotic protein Bcl-2-like protein 1 (Bcl-xL) decreased (Fig. 4C). These results suggested that in HL cells, Ag85B activated both the caspase-dependent extrinsic and intrinsic apoptotic pathways.

### Human HL cells have dysfunctional mitochondria after Ag85B treatment

**Figure 5:**
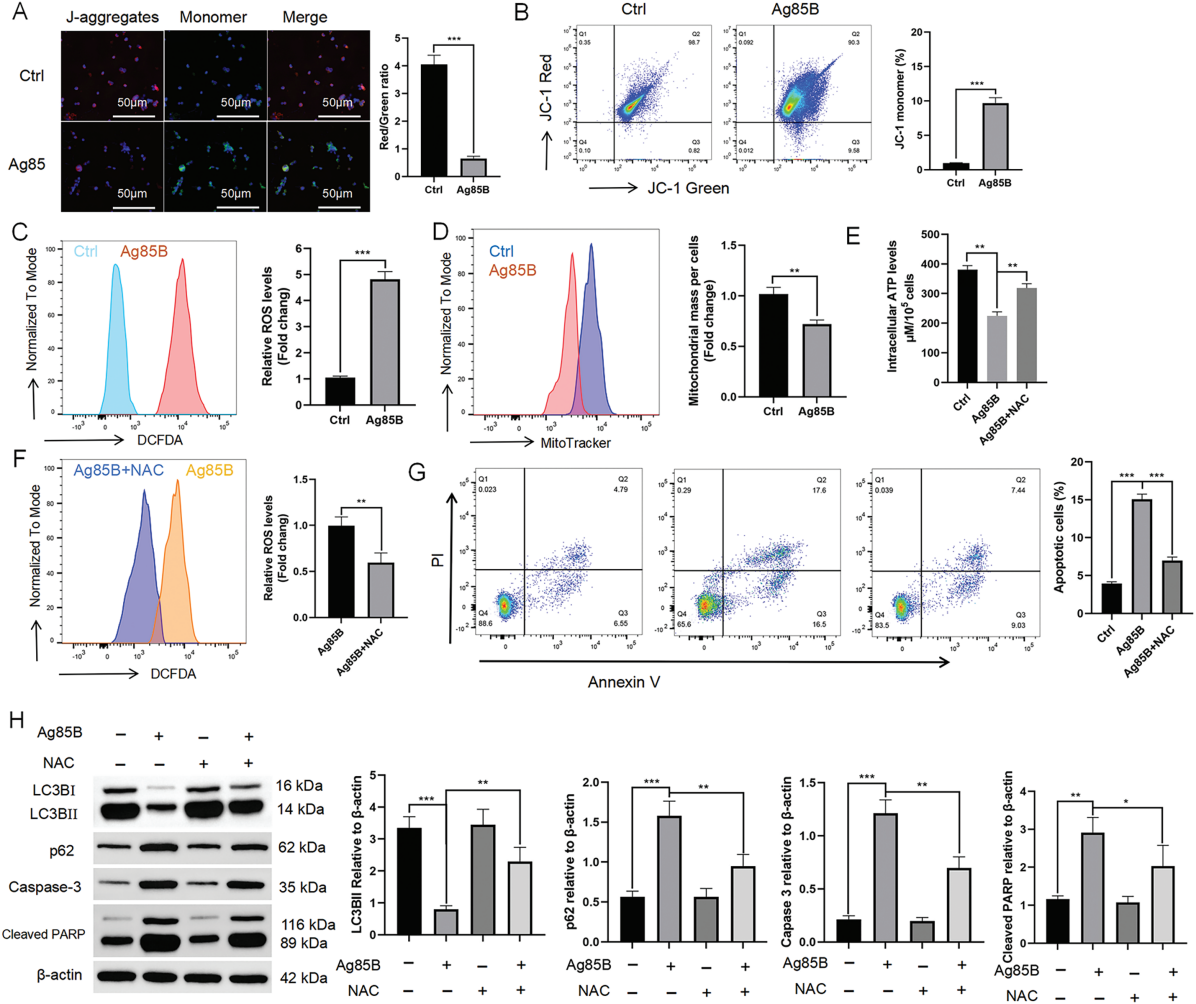
Ag85B-induced autophagy inhibition and apoptosis are associated with mitochondrial reactive oxygen species (ROS) generation. A. Fluorescent probe JC-1 staining of L-428 cells was followed by detection of the membrane potential (MMP) via fluorescence microscopy. Scale bars = 50 μm. B. Flow cytometry detection of the MMP. The histogram shows the percentage of the JC-1 monomers. C. Ag85B was used to treat L-428 cells for 24 h, followed by incubation with 20 μM dichloro-dihydro-fluorescein diacetate (DCFDA) for 30 min, and then the fluorescent intensity was measured. The histograms show the ROS-related mean fluorescence intensity. D. Mitochondrial mass was detected via MitoTracker™ Green FM. E. ATP levels in L-428 cells. F. Flow cytometry detection of ROS levels in L-428 cells treated/not treated with N-acetylcysteine (NAC) at 10 mM. G. Flow cytometry detection of apoptosis in L-428 cells under the indicated conditions. H. Immunoblotting detection of autophagy- and apoptosis-related proteins in L-428 cells. Data represent the mean ± SEM from 2–3 independent experiments (n = 6). *p < 0.05, **p < 0.01, ***p < 0.001. Ctrl, control; LC3B, microtubule associated protein 1 light chain 3 beta; p62, sequestosome 1; PARP, poly (ADP-ribose) polymerase.

Mitochondrial dysfunction is a critical factor implicated in the regulation of cell proliferation, and ROS, predominantly generated by mitochondria, are essential in this process [23]. To assess the MMP, we utilized the fluorescent probe JC-1. Our results indicated that treatment with Ag85B led to a concentration-dependent decrease in aggregated JC-1 (red fluorescence) and a corresponding increase in monomeric JC-1 (green fluorescence) in human HL cells (Fig. 5A,B). These findings suggested that Ag85B might induce the release of ROS from mitochondria. To substantiate this, we employed the fluorescent probe DCFDA to quantify ROS levels, which demonstrated an increase in ROS generation upon exposure to Ag85B in HL cells (Fig. 5C). Thus, we revealed the involvement of mitochondrial ROS in the cellular responses to Ag85B, and the findings warrant further investigation into the molecular mechanisms underlying these observations.

Mitochondrial biogenesis, a process reflecting the cellular capacity for energy production and cellular respiration, was assessed using MitoTracker labeling, a well-established fluorescent probe for live-cell imaging of mitochondria. Our findings indicated a reduction in mitochondrial mass in Ag85B-treated HL cells, as evidenced by decreased MitoTracker fluorescence intensity (Fig. 5D). Additionally, intracellular ATP levels, a critical indicator of mitochondrial function, were significantly diminished following Ag85B administration (Fig. 5E). Together, these findings suggested that Ag85B causes mitochondrial dysfunction in HL cells, potentially through the promotion of ROS release.

Next, we studied the function of ROS in Ag85B-induced autophagy and apoptosis using the antioxidant agent N-acetylcysteine (NAC). NAC pretreatment could block the excessive ROS production induced by Ag85B (Fig. 5F). Flow cytometry analysis showed that NAC attenuated apoptosis and increased ATP levels in HL cells treated with Ag85B (Fig. 5E,G). Western blotting also indicated that NAC ameliorated Ag85B-induced autophagy inhibition and apoptosis (Fig. 5H). These results suggested that ROS exert a vital function in Ag85B-induced autophagy inhibition and apoptosis in HL cells.

### Ag85B activates PI3K/AKT and MAPK signaling to inhibit autophagy

**Figure 6:**
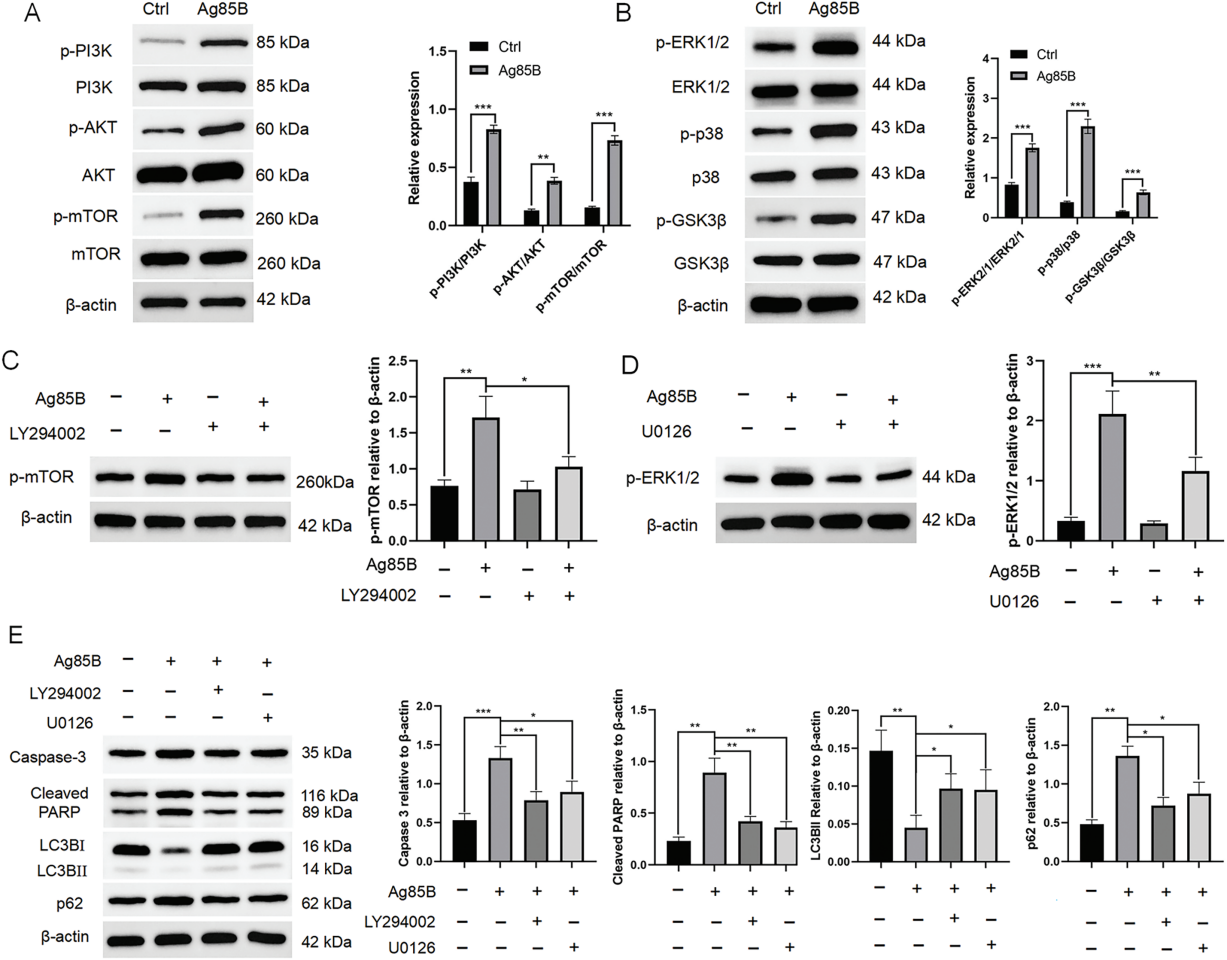
Ag85B activates MAPK and PI3K/AKT pathways. A. Following Ag85B treatment of L-428 for 24 h, immunoblotting was employed to detect PI3K, AKT, mTOR, and their phosphorylated forms (p). B. Immunoblotting detection of the MAPK signaling pathway member protein levels after Ag85B treatment. C, D. L-428 cells were incubated with LY294002 (10 μM) or U0126 (10 μM) for 2 h, and then exposed to Ag85 for 24 h. Western blotting was used to detect the phosphorylation of mTOR and ERK1/2. E. Immunoblotting detection of apoptotic and autophagic marker proteins under the indicated conditions. Data represent the mean ± SEM from two to three independent tests (n = 6). *p < 0.05, **p < 0.01, ***p < 0.001. Ctrl, control; MAPK, mitogen activated protein kinase; PI3K, phosphatidylinositol-4,5-bisphosphate 3-kinase; AKT, protein kinase B; mTOR, mechanistic target of rapamycin kinase; ERK, extracellular regulated kinase; p38, mitogen-activated protein kinase 14; GSK3β, glycogen synthase kinase 3 beta; LC3B, microtubule associated protein 1 light chain 3 beta; p62, sequestosome 1; PARP, poly (ADP-ribose) polymerase.

Mitogen activated protein kinase (MAPK) and phosphatidylinositol-4,5-bisphosphate 3-kinase (PI3K)/protein kinase B (AKT) signaling pathways (negative regulators of autophagy) are key factors that mediate autophagy and apoptosis interactions in tumor cells [24,25]. Therefore, we detected the expression of proteins related to the two pathways. Ag85B induced significant phosphorylation (activation) of MAPK and PI3K/AKT in HL cells (Fig. 6A,B).

To further study whether Ag85B-induced autophagy and apoptosis are related to the two signal pathways, cells were exposed to the PI3K/AKT inhibitor, LY294002, and the extracellular regulated kinase (ERK) inhibitor, U0126, followed by the administration of Ag85B. LY294002 significantly inhibited the Ag85B-induced phosphorylation of mechanistic target of rapamycin kinase (mTOR) in HL cells (Fig. 6C). In addition, U0126 blocked Ag85B-mediated ERK1/2 phosphorylation (Fig. 6D). Meanwhile, we observed that LY294002 and U0126 attenuated the increases in caspase-3 and PARP induced by Ag85B (Fig. 6E). We further analyzed the variations in autophagy of HL cells after blocking the MAPK and PI3K/AKT pathways. LY294002 and U0126 alleviated the inhibition of autophagy induced by Ag85B (Fig. 6E). In conclusion, Ag85B-induced inactivation of autophagy is mediated MAPK and PI3K/AKT pathway activation in HL cells.

### In vivo lymphoma growth is inhibited by Ag85B

**Figure 7:**
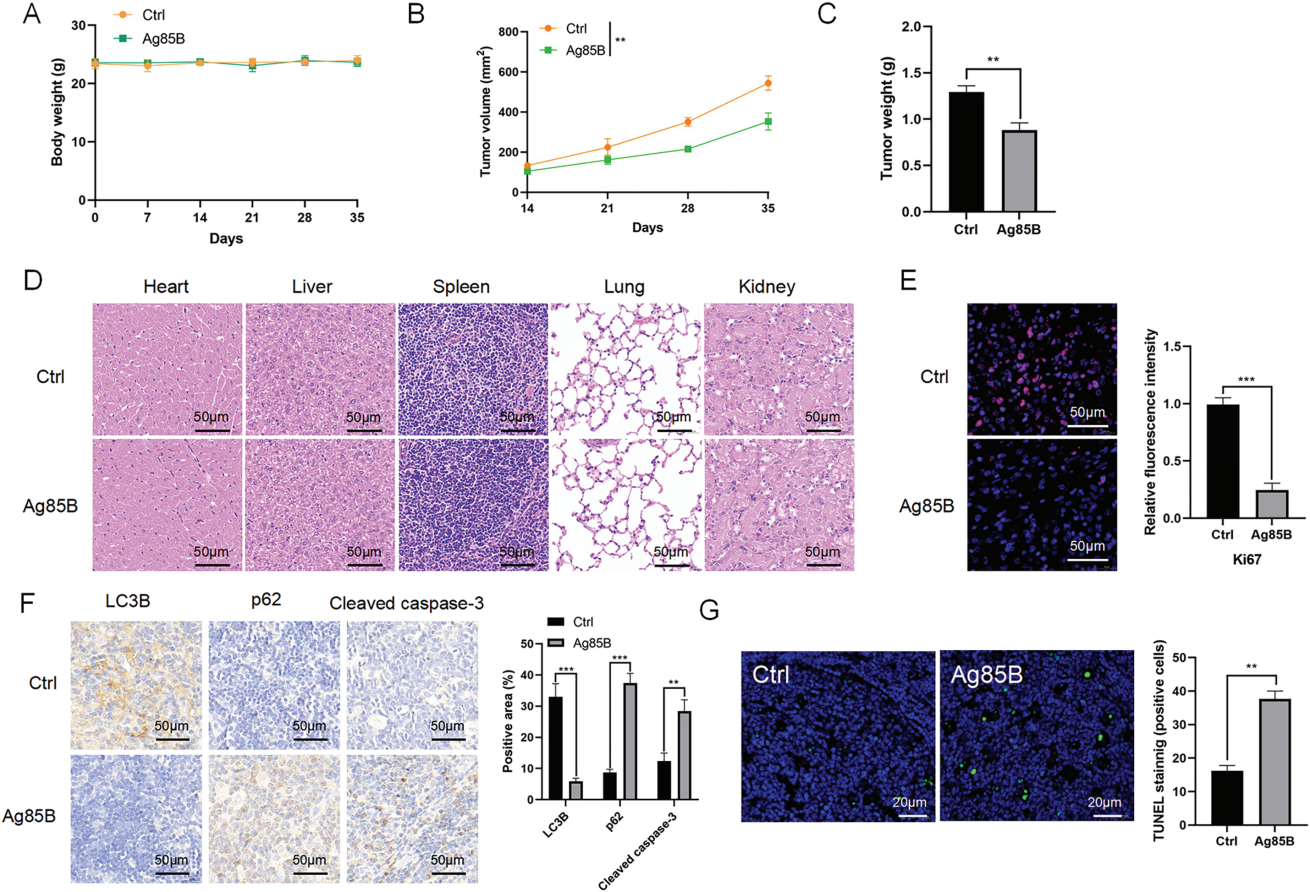
Ag85B inhibits the tumor growth in BALB/c Nude mice xenografted with L-428 cells. A. The body weights of mice under the indicated conditions. B. The tumor volume was detected to draw tumor growth curves. C. Measurement of tumor weights in the different groups. D. H&E staining for the histological evaluation of vital organs. Scale bars = 50 μm. E. Representative immunofluorescence images of Ki-67 staining of tumor samples. Scale bars = 50 μm. F. Representative immunohistochemical images of LC3B, p62, and cleaved caspase-3 staining of tumor samples. Scale bars = 50 μm. G. Apoptotic tumor cells were detected by TUNEL staining. Scale bars = 20 μm. Apoptotic cell numbers were assessed utilizing ImageJ software. Data are shown as the mean ± SEM from two to three independent tests (n = 6). **p < 0.01, ***p < 0.001. Ctrl, control; Ki-67, marker of proliferation Ki-67; LC3B, microtubule associated protein 1 light chain 3 beta; p62, sequestosome 1.

Ag85B’s anti-tumor activity was then assessed *in vivo* using BALB/c Nude mice xenografted with L-428 cells. The body weights of the Ag85B-treated mice did not change significantly (Fig. 7A). The main organs of the Ag85B-treated mice did not show any significant damage according to H&E staining (Fig. 7D). Ag85B significantly inhibited the growth of tumors *in vivo* (Fig. 7B,C). These results are consistent with the significant reduction in Ki-67 levels in L-428 tumors (Fig. 7E), suggesting that Ag85B could effectively inhibit tumor cell proliferation. We also investigated the expression of LC3B and p62 using immunohistochemistry. Ag85B reduced the LC3B levels and increased p62 levels in L-428 tumors. (Fig. 7F). Cleaved caspase-3 levels markedly increased in the L-428 tumors of Ag85B-treated mice (Fig. 7F). Additionally, Ag85B administration increased tumor cell apoptosis and necrosis (Fig. 7G). Thus, Ag85B treatment represents a safe way to exert antitumor effects *in vivo*.

## Discussion

Germinal center B cells are the origin cells of HL, and autophagy plays a key role in the pathogenesis of HL [10]. While conventional treatment strategies for HL, such as radiation therapy, chemotherapy, and hematopoietic stem cell transplantation, are often deemed highly effective and curative for the majority of HL cases, a subset of patients remains refractory to frontline regimens. Additionally, many individuals rendered disease-free by these treatments still face premature mortality because of the long-term toxic effects of therapy. These realities underscore an urgent clinical need for treatments that can achieve high efficacy with minimized toxicity. Autophagy has emerged as a promising new therapeutic target in HL, garnering substantial research attention. However, the efficacy of current autophagy inhibitors, including hydroxychloroquine and chloroquine, remains uncertain. As a result, they have not yet been integrated into HL research. This highlights the pressing need to discover novel and potent autophagy inhibitors that could offer improved treatment options for patients with HL. Ag85, as a natural compound with no significant toxicity or side effects, might become a new autophagy inhibitor for the treatment of HL. Herein, we observed increased proliferation and autophagy in human HL primary tissues. Ag85B inhibited autophagy signals by activating of the PI3K/AKT and MPAK pathways. Furthermore, Ag85B exerted anti-tumor activity in HL by inhibiting cell proliferation and promoting cell apoptosis. Using a xenograft mouse model, we also found that Ag85B markedly blocked tumor growth and promoted cell apoptosis.

The mycobacterial antigen Ag85 (Ag85 A, B, or C), was found to induce a protective immune response [16,17]. A previous study indicated that Ag85B induced cell apoptosis by inhibiting autophagy in WI-38 cells [18]. In the present study, we found that Ag85B impairs autophagy, which is the foundation for its anti-tumor effect on HL cells.

Autophagy participates in the pathogenesis of many diseases, such as cardiovascular diseases, atherosclerosis, and cancers [26–30]. Autophagy causes cells to isolate a portion of the cytoplasm and form small vesicles (autophagosomes), which sequester the contents inside the cytoplasm. Subsequently autophagosomes fuse with lysosomes, and the cytoplasm inside is then degraded and its constituents recovered [31]. LC3, the main component of autophagosomes, interacts with p62, which combines with ubiquitinated cargos [31,32]. During autophagy, p62 is constantly degraded. Thus, a decrease in p62 levels indicates an activated autophagy pathway [32,33].

Many tumors have been proven to be associated with autophagy [34,35]. Autophagy can suppress tumor initiation at the early stage of tumor progression, and is associated with tumor survival in the tumor microenvironment, which is characterized by low nutrients, low oxygen, and immune cell attack [36,37]. Autophagy is activated in cancer cells, which can be sensitized to chemotherapy drugs via genetic ablation of autophagy-related genes or pharmacological inhibition of autophagy [38–41]. Inhibition of the autophagy pathway represents a novel strategy to treat malignant tumors in clinical practice. To date, many clinical trials have been performed to analyze the effects of autophagy inhibitors, combined with cytotoxic chemotherapy and targeted drugs, in the treatment of solid and hematological cancers. Early results suggest that autophagy inhibition combined with anti-cancer therapy appears to be safe and can improve the efficacy of different anti-cancer treatments for melanoma, multiple myeloma, and glioblastoma [36,42]. In addition to existing FDA-approved autophagy inhibitors to treat malaria, such as hydroxychloroquine and chloroquine, it is clear that there is a need to identify other novel and efficient autophagy inhibitors. As such, Ag85B seems to be a good candidate to treat HL.

Malignant tumor cells present with cell cycle dysregulation and intense proliferation. Autophagy inhibition can lead to cell cycle arrest and cell death [43]. We found that Ag85B significantly increased the number of HL cells in the S phase. Cyclins and CDKs play a crucial role in regulating the cell cycle [44]. Cyclins can link with the CDK family and regulate their expression, ultimately leading to a marked enhancement of the proportion of S-phase cells [45]. We found that Ag85B downregulated CDK1 and CDK2, and upregulated cyclin A2, B1, and E1. Moreover, cyclin D1 is only inhibited in the S phase, thus representing an indicator for S-phase arrest [46]. In our results, Ag85B reduced cyclin D1 expression. Ag85B also regulated cell cycle-related molecules, such as p53, p21 and c-MYC. Together, the results showed that Ag85B induced S-phase arrest in HL cells possibly through the c-MYC/p53/p21 pathway to regulate CDKs and cyclin expression.

The two main mechanisms for cell programmed death are autophagy and apoptosis, which induce cell death through alternative, complementary, or synergistic mechanisms [47]. Acute myeloid leukemia cell apoptosis is enhanced by autophagy [19], whereas inhibiting autophagy can greatly promote the apoptosis of oral cancer cells [48]. Correspondingly, the present research found that Ag85B induces cell apoptosis and leads to cell death. Moreover, activation of autophagy alleviated Ag85B-induced cell death, suggesting that Ag85B promotes HL cell death in an autophagy-dependent manner. Previous studies indicated that ROS could induce cell apoptosis [49,50]. In our results, on the one hand, Ag85B significantly increased the production of ROS; on the other hand, ROS inhibitors could effectively inhibit cell apoptosis, suggesting that the ROS induced by Ag85B partially contributed to cell death.

The MAPK and PI3K/AKT signaling pathways are intimately related to cell autophagy and apoptosis [51,52]. Importantly, cell growth and survival are regulated by the PI3K/AKT/mTOR pathway, and its dysregulation is often associated with multidrug resistance in cancers [53]. In the context of lymphomas, this pathway has an important function in disease progression and the response to therapy. For instance, in diffuse large B-cell lymphomas (DLBCL), it has been suggested that the PI3K/AKT pathway functions in the regulation of autophagy, which in turn affects the survival of lymphoma cells [54]. By contrast, the MAPK pathway is involved in cell surface-nuclear signaling, exerting a vital function in cellular responses to stress and mitogenic signals. The development and progression of various cancers, including lymphomas, are associated with aberrant MAPK pathway activation [55]. In our study, we observed that Ag85B treatment affects the MAPK and PI3K/AKT pathway, which could have implications for the modulation of autophagy and apoptosis in HL and potentially other cancers. Moreover, the application of two pathway inhibitors, LY294002 and U0126, confirmed that Ag85B induces cell apoptosis and inhibits autophagy via the PI3K/AKT and MAPK signaling pathways. These results provided a foundation for further exploration of its therapeutic potential.

This study had some limitations. Our findings are based on a single cell line (L-428) and a single animal model, which might constrain the broader applicability of our results. To enhance the validity of our conclusions, further studies are warranted, particularly those involving additional HL cell lines and the use of patient-derived xenografts. Note that the proportion of malignant HL cells in the total tumor mass is relatively low, generally below 1%. Therefore, obtaining sufficient purified HRS tumor cells is a challenge.

## Conclusion

Ag85B restrains HL growth by inhibiting autophagy, which might be related to activation of the PI3K/AKT/mTOR and MAPK pathways. These findings might provide theoretical support for the future treatment of HL.

## Data Availability

Data are available from the corresponding author upon reasonable request.
